# The Role of User Control in Adherence to and Knowledge Gained from a Website: Randomized Comparison Between a Tunneled Version and a Freedom-of-Choice Version

**DOI:** 10.2196/jmir.1922

**Published:** 2012-03-09

**Authors:** Rik Crutzen, Dianne Cyr, Nanne K de Vries

**Affiliations:** ^1^Department of Health PromotionMaastricht University/CAPHRIMaastrichtNetherlands; ^2^Beedie School of BusinessSimon Fraser UniversitySurrey, BCCanada

**Keywords:** website use, user control, user perceptions, Internet, interventions

## Abstract

**Background:**

Internet-delivered interventions can effectively change health risk behaviors and their determinants, but adherence to these interventions once they are accessed is very low. Therefore, it is relevant and necessary to systematically manipulate website characteristics to test their effect on website use. This study focuses on user control as a website characteristic.

**Objective:**

To test whether and how user control (the freedom of choice to skip pages) can increase website use and knowledge gained from the website.

**Methods:**

Participants older than 18 years were drawn from the Dutch Internet population (in June 2011) and completed a hepatitis knowledge questionnaire. Subsequently, they were randomly assigned to three groups: (1) a tunneled version of the website with less user control; (2) a high user control version of the website where visitors had the freedom of choice to skip pages; and (3) a control group that was not exposed to the website. Participants completed (1) a questionnaire of validated measures regarding user perceptions immediately after exposure to the website (except for the control group), and (2) a hepatitis knowledge questionnaire after one week to test whether participants in the experimental groups only clicked through the website or actually processed and learned its content. Server registrations were used to assess website use. Analyses of covariance (ANCOVA) using all available data were conducted to determine whether user control increases website use. Structural equation models (SEM) using all available data were constructed to test how user control increases website use—a latent variable derived from number of pages visited and time on website.

**Results:**

Of the 1044 persons invited to participate, 668 took part (668/1044, 64.0%). One half of participants (332/668 49.7%) were female and the mean age was 49 years (SD 16). A total of 571 participants completed the one-week follow-up measure regarding hepatitis knowledge (571/668, 85.5%). The findings demonstrate that having less user control (ie, a tunneled version of the website) had a negative effect on users’ perception of efficiency (F_1,452_ = 97.69, P < .001), but a positive effect on number of pages visited (F_1,452_ = 171.49, P < .001), time on the website (F_1,452_ = 6.32, P = .01), and knowledge gained from the website (F_1,452_ = 134.32, P < .001). The direct effect of having less user control appeared to surpass the effect mediated by efficiency, because website use was higher among participants exposed to the tunneled version of the website in comparison with those having the freedom of choice to skip pages.

**Conclusions:**

The key finding that visitors demonstrated increased website use in the tunneled version of the website indicates that visitors should be carefully guided through the intervention for future intervention websites.

## Introduction

Internet-delivered interventions can effectively change health risk behaviors and their determinants [1,2], but the actual use of these interventions by the target group once they access the website is very low [3,4]. For example, server statistics of a web-based intervention promoting heart-healthy behaviors showed that 285,146 visitors from unique IP addresses accessed the home page over a 36-month period, but 56.3% of visitors left the intervention website within 30 sec [5]. This finding touches upon the critical issue in Internet-delivered interventions: How can these interventions have a public health impact if people use the actual intervention so briefly? Therefore, it is relevant and necessary to focus on factors related to use of an intervention once people arrive at the intervention website (ie, website use) [6]. These factors relate to the *visitor* (eg, people’s motivation to be healthy [7,8]) as well as the *intervention* website (eg, offering tailored information [9-11]).

The content of the website is important [12], but the specific characteristics of the website itself are also important. A previous study stressed the need for future research to systematically manipulate website characteristics and, subsequently, to link these manipulations to website use [13]. The current study follows this recommendation and focuses on user control as a website characteristic.

User control covers the voluntary and instrumental actions of the website visitor [14,15]. This is an important characteristic of a website that shapes the two-way online interaction and the exchange of information with website visitors resulting in a user experience [16,17]. User experience refers to what a person thinks and feels during and after exposure to a website [18]. The main idea is that a positive user experience increases website use. User experience consists of cognitive perceptions and affective perceptions [19]. Cognitive perceptions are rational in nature and induced by utilitarian or cognitive motives. Affective perceptions are emotional in nature and induced by hedonic or affective motives [20].

The key user perceptions are efficiency, effectiveness, enjoyment, and active trust [19]. These terms are derived from other fields, such as e-commerce. Although they can have a different meaning within public health, we chose to use the same terminology as in previous studies for consistency and to avoid confusion. *Efficiency* refers to easy search and access of information provided; *effectiveness* refers to the quality of that information (eg, in terms of relevance) [21]. These cognitive perceptions have parallels with perceived ease of use and perceived usefulness in the technology acceptance model, but are applicable in a broader context [22]. The idea that a positive user experience increases website use does not only apply to cognitive perceptions, but also to affective perceptions [23]. These affective perceptions are often referred to as *enjoyment* [24]. *Active trust* refers to the confidence in acting on the provided information on a website, which can result in increased website use [25]. The previous study that served as the basis for the present one consistently demonstrated that effectiveness and enjoyment both had a positive effect on intention to use, which was mediated by active trust [13]. Efficiency did not have an effect, but this could be explained by the goal of the websites being used in that study, which were aimed purely at behavioral change instead of providing information only (such as the websites used in the present study). Therefore, in this study, efficiency, effectiveness, and enjoyment were expected to increase website use (Hypothesis 1), which was expected to be mediated by active trust (Hypothesis 2).

To gain more insight into *how* user control can help to increase website use, it is necessary to study the effect of user control on user perceptions. Since user control provides the ability to explore and to understand the structure of a website [26,27], it allows visitors to be involved in the cognitive processing of information. This is closely related to the concept of efficiency (ie, “easy search and access of information”) and indicates a certain level of user involvement. Previous research revealed that the positive effect of freedom of choice (ie, high user control) on preference regarding websites was mediated by efficiency [28].

One of the most common issues for websites is the lack of user control [29], which might lead to a reactance effect [30]: A constrained freedom of choice results in a negative effect on preference for that website [28]. The visitor, however, can still decide whether or not to keep using the website. When a visitor has the freedom to decide whether to use the website, but at the same time has less influence on how to use it, this can be interpreted as a form of libertarian paternalism. To elaborate, this is a weak form of paternalism that guides people (eg, a tunneled version of a website with less user control) without necessarily restricting their choices (eg, the decision to keep using a website) [31]. The crux of libertarian paternalism is that by allowing choice, but designing it in such a way that skews outcomes toward particular directions (eg, increased website use), the visitor experiences a degree of free will [32]. Hence, user control was hypothesized to increase efficiency [33,34] (Hypothesis 3), but to decrease actual website use, because it is the opposite of libertarian paternalism (Hypothesis 4).

**Figure 1:**
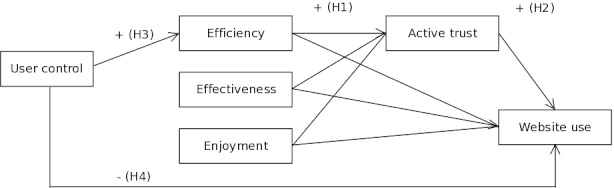
Conceptual model with four hypothesized paths (H1–H4).

Hypothesis 4 may seem in opposition to Hypothesis 3: Although user control was expected to decrease website use, it was expected to increase efficiency. To answer the question *whether* user control can help to increase website use, we explored whether the possible direct effect of user control surpasses the possible effect mediated by efficiency. Figure 1 provides an overview of the conceptual model used in this study.

## Methods

The experimental condition for this study was a website about hepatitis A, B, and C virus (HAV, HBV, and HCV) infections. These infections all affect the liver, but each of the HAV, HBV, and HCV infections differs in terms of mode of transmission, consequences, and prevention. The website consisted of a home page plus four pages of information per hepatitis virus. The first page introduced the virus briefly and the other three pages outlined information about transmission, consequences, and prevention, respectively. This resulted in a total of twelve pages of website content (including all virus types) plus the home page. The content for these pages was based on information from the Dutch National Hepatitis Centre (eg, information leaflets) and was limited to mode of transmission, consequences, and prevention of HAV, HBV, and HCV infections. The content was text-based, purely informative, non-tailored, and very brief (ie, 5–10 lines of text per page).

### Design and Procedure

**Figure 2:**
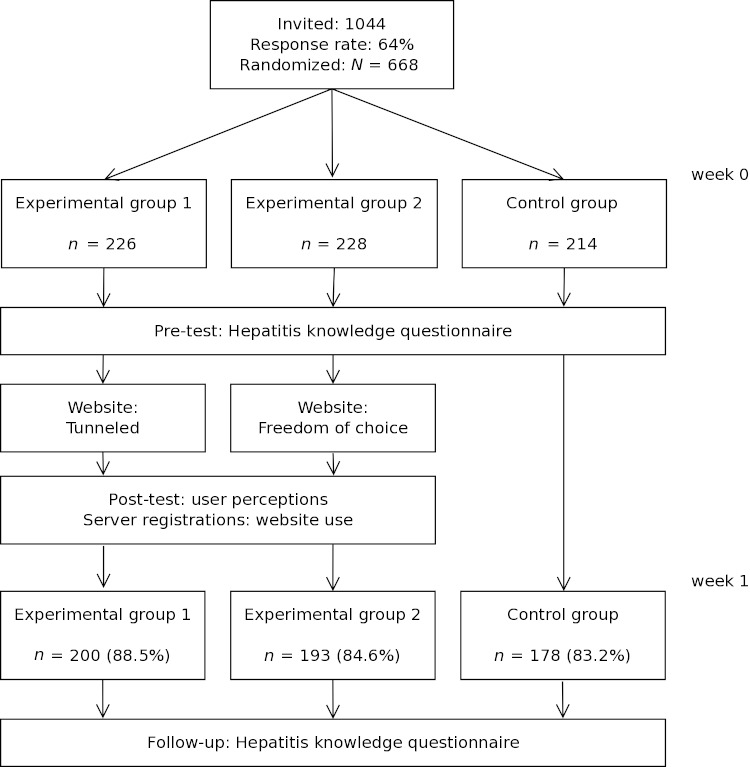
Flowchart of study design and attrition.

Participants were randomly assigned to three groups (Fig 2), but were not informed about the existence of these three groups or that the focus of the study was on website use. Two groups were experimental groups in which user control was manipulated. In experimental group 1 (ie, tunneled group), participants viewed a tunneled version of the website [35]; in experimental group 2 (ie, freedom of choice group), participants had freedom of choice (eg, they could skip pages [36]). The third group was a control group who were not exposed to the website.

For the tunneled group, the web pages could be viewed only in a pre-determined order (introduction of the virus, transmission, consequences, and prevention for HAV, HBV, and HCV, respectively) and pages could not be skipped. This is in-line with libertarian paternalism: allowing choice to stop using the website, but designing it to skew toward increased website use. The number of pages and the content of both website versions for both experimental groups were identical. There was no human involvement (eg, health professional assistance); the website was fully automated. The third group was a control group that was not exposed to the website, but which was added to test whether participants in the experimental groups just clicked through the website or actually processed and learned its content (ie, by comparing between-group differences regarding hepatitis knowledge at follow-up). Participants in the control group were only required to complete the pre-test and follow-up measurements. Their access to other websites related to hepatitis was unable to be controlled during the study period due to participants completing the study remotely.

All three groups—the control group and the experimental groups before exposure to the website—took an initial questionnaire to establish their baseline hepatitis knowledge (“pre-test”). All measures are described in the measurements section. After the pre-test, the two experimental groups were directed to their assigned version of the website and participants knew they had to give their opinion about the website afterwards. Participants were asked to base their opinion about the website on their first impression and were told they could freely explore the website until they started completing the post-test immediately upon leaving the website. The objective was to prevent participants from thoroughly studying the website, and to mimic a real-life situation in which time and opportunity to invest in the website is limited [5].

The two experimental groups took another test immediately after exposure to the website (“post-test”) measuring user perceptions of the website and their perceptions of user control as a manipulation check. For these measures, it was stressed that there were no right or wrong answers. There was no post-test for the control group, since they were not exposed to the website.

One week later, participants were invited to complete the follow-up measure, which was a hepatitis knowledge questionnaire similar to the pre-test. The study was conducted in June 2011 and participants could complete the study at their own convenience (eg, at their own home). Participants received an incentive (ie, credit points for research panel members; as explained in the Participants section) to participate in the study, which represented a value of €1.39. Panel members can save credit points over time, which can be exchanged for online vouchers valid in several stores in the Netherlands. Relevant ethical safeguards regarding Dutch law were met for participant confidentiality and consent.

### Participants

Participants were recruited through a research panel of a Dutch Internet research agency [37]; therefore, they could be considered computer literate. From this panel, a stratified sample of 1044 potential participants were invited to participate in a study about hepatitis through email. Informed consent was obtained online (ie, the regular procedure for this research panel). This sample was representative of the Dutch Internet population above 18 years, taking into account gender, age, and level of education. Of those invited, 668 participated in the study (668/1044, 64.0%). Half of the participants (332/668, 49.7%) were female and the mean age of participants was 49 years (SD 16). Of the participants, 35.5% had a low level of highest completed education (equivalent to primary school/junior high school), 38.2% an intermediate level (equivalent to senior high school/junior college), and 26.3% a high level (equivalent to college/university). Those 668 that participated were invited to complete the follow-up measure and 571 of them did so (571/668, 85.5%). There was no selective dropout regarding gender (χ^2^ = 1.3, *P* = .25), age (*F*_1,666_ = 0.08, *P* = .77), level of education (χ^2^ = 1.3, *P* = .52) or hepatitis knowledge at baseline (*F*_1,666_ = 3.53, *P* = .06). Furthermore, dropout did not differ between the three groups (χ^2^ = 2.7, *P* = .26).

### Measurements

#### Hepatitis Knowledge Questionnaire

Fifteen true/false items (including a “don’t know” option) about transmission, consequences, and prevention of HAV, HBV, and HCV infections were used to assess hepatitis knowledge. The sum score of all correctly answered items was used in the analyses. The correct answer to these items was available on the website, but were only communicated to the participants after the study. The items concerned “how-to” knowledge: practical knowledge concerning how to do something [38].

#### Website Use

Server registrations were used to assess website use [39,40] which was operationalized by the number of pages visited (ranging from 0 to 12) [41]. Furthermore, time on the website was tracked to detect whether participants simply clicked from one page to the next, which would artificially boost the number of pages visited.

#### User Perceptions

Efficiency (Cronbach alpha = .94), effectiveness (Cronbach alpha = .90), enjoyment (Cronbach alpha = .97), and active trust (Cronbach alpha = .88) were assessed by three items each. Participants answered questions such as “I was able to access the information quickly on this website” (efficiency), “the website provided me with relevant information about...” (effectiveness), “I found my visit to this website enjoyable” (enjoyment), and “I would act upon the information presented on this website if needed” (active trust) using a 7-point Likert scale ranging from “strongly disagree” (= 1) to “strongly agree” (= 7).These measures were previously used and validated in the Dutch language [10]. Table 1 shows the correlations between these user perceptions.

**Table 1:** Correlation matrix of user perceptions.

User perception^a^	1	2	3	4
1. Efficiency	-	.55	.52	.56
2. Effectiveness		-	.63	.68
3. Enjoyment			-	.66
4. Active trust				-

^a^ Pearson correlation coefficient has been used as a measure of correlation between user perceptions. All correlation coefficients were significant (*P* < .001).

#### Perceived User Control

Four items measured perceived user control (Cronbach alpha = .79) as a manipulation check [42]. Items such as “while I was on the website, I could choose freely what I wanted to see” were answered on a 7-point Likert scale ranging from “strongly disagree” (= 1) to “strongly agree” (= 7).

### Analyses

First, using Predictive Analytics SoftWare Statistics 18.0 (International Business Machines Corporation, Armonk, NY), analyses of covariance (ANCOVA) using all available data were conducted (1) to test whether the manipulation was successful; (2) to test whether there were group differences regarding hepatitis knowledge at follow-up, taking into account hepatitis knowledge at the pre-test [43]; and (3) to test the direct effect of user control on number of pages visited, time on the website (ie, whether user control increases website use), and user perceptions. Number of pages visited and time on the website were square root transformed to meet assumptions of normality.

Second, using Mplus 5 (Muthén & Muthén, Los Angeles, CA), structural equation models (SEMs) using all available data were constructed to test the hypothesized conceptual model (ie, how user control increases website use). Efficiency was regressed on user control. Website use—a latent variable made up from number of pages visited and time on the website—was regressed on user control, efficiency, effectiveness, enjoyment, and active trust. Active trust was regressed on efficiency, effectiveness, and enjoyment. Subsequently, (1) non-significant paths were left out of the conceptual model for the sake of parsimony, and (2) additional paths were added to the conceptual model based on significant modification indices. The latter was done to explore whether unanticipated relationships might explain variance in website use (which was not the case). A level of significance of *P* = .05 was used for the relationships within the model.

Model fit indices used were the comparative fit index (CFI), the Tucker-Lewis index (TLI), the root mean square error of approximation (RMSEA), and the standardized root mean square residual (SRMR). Both CFI and TLI are goodness-of-fit indices where larger values signal better fit. Values over .95 indicate close fit. The RMSEA and SRMR are goodness-of-fit indices where larger values signal worse fit. Indicators of close fit are, respectively, RMSEA ≤ .05 and SRMR ≤ .09 [44,45].

## Results

Perceived user control is higher (5.2 vs 3.9 on a 7-point Likert scale) if participants had freedom of choice (eg, could skip pages) instead of a tunneled version of the website (*F*_1,452_ = 134.32, *P* < .001), indicating that the manipulation of user control was successful. Table 2 shows hepatitis knowledge per group, both at the pre-test and follow-up. There are group differences regarding hepatitis knowledge at follow-up, after controlling for hepatitis knowledge at the pre-test (*F*_2,567_ = 47.24, *P* < .001). All pairwise comparisons are significant (*P* < .001) indicating that participants in the tunneled group score higher on hepatitis knowledge compared with the freedom of choice group. Both experimental groups score higher on hepatitis knowledge in comparison with the control group, indicating that participants do not only click through the website, but actually process and learned the content.

**Table 2:** Pre-test and follow-up hepatitis knowledge scores.

Group	Pre-test^a^	Follow-up^a^
	Mean (SD)	Mean (SD)
Tunneled group (*n* = 200)	5.0 (3.3)	8.2 (3.5)
Freedom of choice group (*n* = 193)	5.4 (3.1)	7.2 (3.5)
Control group (*n* = 178)	5.4 (3.2)	5.6 (3.3)

^a^ Maximum score is 15 points.

Table 3 shows that having a lesser degree of user control has a negative effect on efficiency, but a positive effect on number of pages visited (confirming Hypotheses 3 and 4). Participants do not simply click from one page to the next, since the time on the website is also longer in the tunneled group (3:50 min) compared with the freedom of choice group (2:38 min) (*F*_1,452_ = 6.32, *P* = .01).

**Table 3:** The direct effect of user control on number of pages visited and user perceptions.

		Tunneled group (*n* = 226)	Freedom of choice group (*n* = 288)		
Measure	Range	Mean (SD)	Mean (SD)	*F*_1,452_	*P* Value
Number of pages visited	0–12	11.4 (2.3)	7.4 (4.0)	171.49	< .001
Efficiency	1–7	4.8 (1.7)	6.1 (1.1)	97.69	< .001
Effectiveness	1–7	5.8 (1.2)	5.8 (1.1)	0.56	.46
Enjoyment	1–7	4.9 (1.5)	5.0 (1.4)	0.72	.40
Active trust	1–7	5.1 (1.4)	5.3 (1.4)	4.15	.04

**Figure 3:**
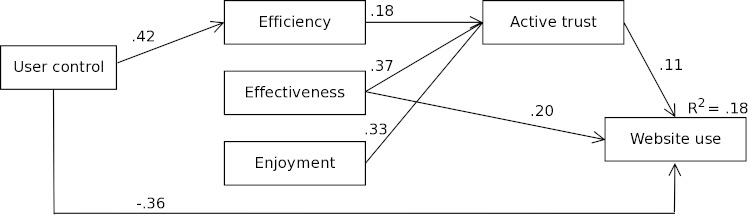
Final model including standardized betas of significant paths within the model.

Figure 3 illustrates the final structural equation model. User control has a positive effect on efficiency, but a negative effect on website use. The direct effect appears to surpass the effect mediated by efficiency because website use is higher in the tunneled group compared to the freedom of choice group. These findings also support Hypotheses 3 and 4. Efficiency has a positive effect on website use, but effectiveness and enjoyment do not have a direct effect on website use (only partly confirming Hypothesis 1). Therefore, these paths were removed from the final model. Active trust, however, mediates the relationship between efficiency, effectiveness, enjoyment, and active trust (confirming Hypothesis 2). Based on modification indices, no paths were added to the conceptual model, implying that user control is only related to efficiency and website use. The CFI and TLI are .97 and .96, respectively; RMSEA and SRMR are .08 and .03, respectively. All of these fit indices indicate a close fit for the final model.

## Discussion

A key finding of this study is that user control does not help to increase website use, which leads to a smaller effect on knowledge gain in comparison with the tunneled version of the website. Although visitors thought that having control made it easier to search and access information, this was negated by the direct negative effect that user control had on website use. In short, user control decreases website use. The increase in perception of efficiency, however, is in-line with the *idea* of freedom of choice as more important than its actual existence [46]. These findings indicate that, for future intervention websites, visitors should be carefully guided through the intervention (ie, less user control).

As an element of interaction design (ie, options involved in performing and completing tasks), it is proposed user control should fit the task of the user and the objectives of website accessed [18]. For example, if a goal for a website is to encourage visitors to review all pages about self-management, then it would be useful for visitors to be guided through these pages. However, if the website is to serve as a data bank or encyclopedia that visitors may consult, then it would be more useful to add a search function and an effective menu structure [47].

Most of our hypotheses regarding user perceptions are confirmed and are in-line with the previous study [13]. Active trust mediates the positive effects of efficiency, effectiveness, and enjoyment on website use. The lack of a direct effect of enjoyment on website use was unanticipated. Based on previous research, an explanation could be that enjoyment is related to cognitive perceptions [48], and website use is fully mediated by active trust. Thus, cognitive perceptions might be a catalyst for the positive effect of enjoyment on website use. The lack of a direct effect of efficiency on website use is puzzling. Since previous research demonstrated that active trust is a mediating variable associated with intention to use a website [49,50], it might be that active trust reduces the possible direct impact of efficiency since the explained variance of active trust overlaps with the explained variance of efficiency. Future research is needed to investigate the plausibility of this explanation.

Another avenue for future research is to examine factors related to the visitor given that the direct effect resulting from the manipulation of a website characteristic (ie, user control) surpassed the effect mediated by user perceptions (ie, efficiency). Hence, it is worthwhile to investigate whether the impact of website characteristics is greater for certain visitors than for others. Knowing something about the personalities of those who favor certain website characteristics will provide better insight into factors behind website use [51]. Ross and colleagues [52] took a first step in this direction by linking personality factors to the use of Facebook features.

Contrary to previous research [13,19,33], a positive characteristic of this study is that actual website use was tracked and measured instead of using self-reported data only. More specifically, website use was tracked by means of server registrations, which in contrast to self-reports, is independent of visitors’ memory, interpretation, or social desirability [53,54]. Moreover, within the setting of a research panel in which participants could complete the study at their own convenience (eg, at their own home), a real-life situation has been mimicked. Participants could freely explore the website, without the limitations of a laboratory setting (eg, standardized environment, forced exposure), which enhanced the validity of the study (ie, in vivo versus in vitro testing). Finally, there was a relatively small dropout rate between pre-test and follow-up in this study, which was neither selective dropout nor differed between groups. Hence, there is still good variation in gender, age, and level of education of the participants to warrant generalizability of the findings to the Dutch Internet population above 18 years.

Finally, there are two additional points that evolved from this investigation. First, knowledge regarding hepatitis increased in both experimental conditions compared to the control group. This implies the hepatitis knowledge questionnaire is appropriate to assess whether participants processed and learned content of the website. This is the case even though participants were not necessarily looking for information on hepatitis, which is essential in primary prevention websites aimed at the general public. Nevertheless, it could be that participants were highly interested in hepatitis, since they agreed to participate in a study about this topic. The low scores regarding hepatitis knowledge at pre-test, however, do not support this possible explanation. Second, knowledge increased more in the tunneled group in which website use was higher. Since user control is not directly related to retention [55], this suggests website use increases the likelihood of changes in determinants of health risk behaviors—which is important from a public health point of view. Thus, as an element of interaction design, user control should be carefully considered during the development of Internet-delivered interventions. Our findings indicate future interventions should carefully guide visitors through the website (ie, less user control) to increase website use and subsequently elevate the public health impact of these interventions.

## Abbreviations

ANCOVA: analyses of covariance
CFI: comparative fit index
HAV: hepatitis A virus
HBV: hepatitis B virus
HCV: hepatitis C virus
RMSEA: root mean square error of approximation
SEM: structural equation models
SRMR: standardized root mean square residual
TLI: Tucker-Lewis index
